# Supplementary material for: Prediction rigidities for data-driven chemistry
Source: Faraday Discuss. 2024 Aug 23;256:322–44. doi: 10.1039/d4fd00101j (PMC11423580; doi:10.1039/d4fd00101j)
Supplement: FD-256-D4FD00101J-s001 [file FD-256-D4FD00101J-s001.pdf]

Supplementary Information (SI) for Faraday Discussions.
This journal is © The Royal Society of Chemistry 2024

# Supplementary information for "Prediction rigidities for data-driven chemistry"

Sanggyu Chong, Filippo Bigi, Federico Grasselli, Matthias Kellner, and Michele Ceriotti*
*Laboratory of Computational Science and Modeling, Institute of Materials, École Polytechnique Fédérale de Lausanne, 1015 Lausanne, Switzerland*

## I. ML MODEL TRAINING DETAILS FOR SECTION 3

The SOAP-BPNN models were trained using the implementation found in `metatensor-models`. [1] The following hyperparameters were used for comuting the SOAP descriptors: $r_c = 5.0$ Å, $n_{\max} = 9$, $l_{\max} = 9$, $\sigma_{\text{atom}} = 0.3$ Å, a shifted cosine function with width = 0.5 Å, and a radial scaling function devised by Willatt et al. [2] with rate = 1.0, scale = 2.0 Å, and exponent = 7.0. The Behler-Parrinello NN was set up with 2 hidden layers and 64 neurons per layer. Training was conducted with 32 as the batch size. The Adam optimizer [3] was used with an initial learning rate of 0.001. A `ReduceLROnPlateau` learning rate scheduler implemented within PyTorch [4] was used with a scheduler patience of 5 and reduction factor of 0.75. Early stopping was performed with a patience of 50. Note that the models were trained on intensive quantities of total energies per atom. The PaiNN models were employed with the default hyperparameters of the model for QM9 provided in the `SchNetPack` tutorials. [5] Batch size was set to 100, and the Adam optimzer was used with an initial learning rate of 0.0005. `ReduceLROnPlateau` learning rate scheduler was used with a scheduler patience of 20, and reduction factor of 0.5, and cool-down of 5 epochs. PaiNN was trained on the extensive total energy targets.

The MACE models were used with the following hyperparameters: $r_c = 5.5$ Å, hidden irreps of `128x0e + 128x1o`, [6] and the default values for the rest of the parameters. Both energies and forces were incorporated into the model loss function. Energy loss was defined to be per atom RMSE. Zero of the energy was set to be the isolated atom energy of silicon. Exponential moving average (EMA) and stochstic weight averaging (SWA) techniques were employed in the model training. Model training was performed in two stages: in the first stage, training was performed with an energy weight of 1 and force weight of 100. Adam optimizer was used, and the initial learning rate was set to be 0.01. EMA technique is employed, and an early stopping patience of 100 was used along with a `ReduceLROnPlateau` learning rate scheduler with a scheduler patience of 10 and reduction factor of 0.8. When early stopping was called, the model training entered the second stage, where the model was trained for 100 additional epochs with an energy weight of 1000, force weight of 100, and with the SWA technique employed. Here, the learning rate was fixed at 0.001. Final model is the one taken at the end of the second stage of model training.

SOAP-BPNN and PaiNN were trained on the QM9 dataset. Dataset sampling was done from a refined dataset of 130,831 molecules that excludes the problematic structures flagged by the authors. 10,000 moleculse were randomly chosen as the training set, 1000 as validation, and 1000 as test sets. In training additional models to gather statistics on the local energy variances, 10-fold cross-validation datasets were created with the 10,000 molecules, then only the subsampled training sets were taken, and the validation set was fixed to be the original one. In the case of MACE models, the silicon decamers (10-mers) were generated using a simple algorithm for random cluster growth. The algorithm is iterative, where in each iteration, a "growth" is attempted by randomly selecting an atom from the already-grown cluster and attaching another silicon atom along a random direction with a bond length between 2.0 and 2.5 Å. The move is only accepted if the minimum distance criterion of 2.0 Å between all cluster atoms are satisfied, and any given atom is bonded with no more than 6 neighboring atoms. *Ab initio* reference energy calculations were conducted using `FHI-AIMS` software [7] under "tight" settings and with the PBEsol exchange-correlation functional.[8] Gaussian smearing with $\sigma$ of 0.05 eV was employed. 8000 training, 1000 validation, and 1000 test samples were acquired. The same procedure described above for the QM9 dataset was used to generate the subsampled datasets in studying the local energy variances.

Note that in all cases, we did not fine-tune the model hyperparameters to achieve the best performance possible, as the primary objective of the section was to show the efficacy of the last-layer approximations in obtaining the PR metrics for the NN-based atomistic ML models.

* michele.ceriotti@epfl.ch

## II. ML MODEL TRAINING DETAILS FOR SECTION 4

For the PR-guided dataset augmentation case study, the LE-ACE models were trained with the following hyperparameters: $r_c = 4.5$ Å, $\nu_{\text{max}} = 4$, and the Lapcian eigenstate energy cutoffs of 1000, 300, 200, and 100 eV were used the successive $\nu$ components, for a total number of 908 features. In addition to the smoothness prior of the LE-ACE basis, a regularization $\alpha$ of 1e–5 was used in training the model, and the $\nu$-dependent regularization scheme was turned off. For model training, carbon GAP-17 dataset of Deringer et al. [9] was employed. From the dataset, the liquid and amorphous bulk structures were identified under `config_type` = `bulk_amo`. The surface-containing structures of interest were identified by `config_type` = `surf_amo`. The `bulk_amo` structures were then filtered by their densities, and the structures with $\rho > 3.0$ g/cm$^3$ were taken as the training set for the case study. From the remaining `bulk_amo` structures, 1000 structures were randomly selected and kept as the candidate pool for structure selection. 50 randomly chosen `surf_amo` structures were taken as the target systems of interest for the case study.

For the active learning case study, the LE-ACE model with the following hyperparameters was considered: $r_c = 3.7$ Å, $\nu_{\text{max}} = 3$, and the Lapcian eigenstate energy cutoffs of 1000, 300, 200 eV were used the successive $\nu$ components, for a total number of 775 features. Note that the model was only used to obtain the feature vectors, and no explicit LE-ACE model training was performed. In procuring the dataset, 500 structures were randomly chosen from the entire carbon GAP-17 dataset. [9] The 13,824 atom liquid carbon structure is a randomly sampled snapshot from a MD simulation conducted for an NVT ensemble at 9000 K for 1 ns, using an in-house GAP model trained with `librascal` [10] trained on the same dataset. Hyperparameters for the GAP model is as follows: $r_c = 5.5$ Å, $n_{\text{max}} = 14$, $l_{\text{max}} = 10$, $\sigma_{\text{atom}} = 0.25$ Å, a shifted cosine function with width = 0.5 Å, and a radial scaling function devised by Willatt et al. [2] with rate = 3.0, scale = 2.0 Å, and exponent = 6.0.

## III. DATASET CONSTRUCTION AND ML MODEL TRAINING DETAILS FOR SECTION 5

Both the self-interacting and purified ACE feature vectors were obtained using the `ACEpotentials.jl` and `ACE1x.jl` packages. [11] For both feature vectors, $r_c = 5.5$ Å, $\nu_{\text{max}} = 4$, and total degrees per $\nu$ were set to be 24, 20, 16, and 12, resulting in 929 features in total. We note that setting `pure` = `False` or `True` in using the `ACE1x.ace_basis` function leads to either the self-interacting or purified ACE feature vectors. Model training and CPR calculation were done outside of the Julia-based packages. In training the models, single-value regularization with $\alpha = 0.001$ was performed. The dimer configurations were generated by varying the Si–Si distance from 1.5 to 5.0 Å and uniformly sampling 100 configurations. Trimers, tetramers, and pentamers were generated using the same random growth algorithm explained in Section I, and the *ab initio* energy calculations were conducted in the same manner using `FHI-AIMS`. [7]

For the SOAP+LODE models, all feature vector calculations were performed with `rascaline`. [12] The following hyperparameters and functionalities were used in SOAP: $r_c = 2.8$ Å, GTO radial basis with $n_{\text{max}} = 4$, $l_{\text{max}} = 3$, Gaussian atomic density $\sigma_{\text{atom}} = 0.3$ Å, a shifted cosine function with width = 0.25 Å. The LODE feature vectors were computed with the following hyperparameters: $r_c = 2.8$ Å, monomial basis with $n_{\text{max}} = 4$, $l_{\text{max}} = 3$, a splined LODE density with atomic width = 1.2 Å and potential exponent $p = 3$, and spline accuracy of 1e–10. As explained in the main text, in order to obtain the range-separated SOAP+LODE from non-orthogonal SOAP+LODE, LODE contributions from the atoms within $r_c$ can be subtracted. *Ab initio* reference energy calculations for the water dimer and monomer configurations under the same calculating details outlined in Ref. [13].

## IV. ML MODEL TRAINING AND MD SIMULATION DETAILS FOR SECTION 6

In training the MACE models on coarse-grained water, `SiLU` activation functions within the architecture were all replaced with `tanh` activation functions to induce a more stable learning behavior, especially in dealing with the high level of noise in the force data. $r_c = 6.0$ Å, and hidden irreps were set to be `64x0e + 64x1o`. Batch size of 10 was used. RMS force rescaling implemented with MACE was employed, and the exponential moving average (EMA) technique was applied. Forces-only model training was performed for 50 epochs, and the epoch with the lowest apparent validation loss was taken as the final model. To generate the reference data, classical, non-polarizable force-field molecular dynamics simulations of 128 flexible TIP4P/2005 [14] water molecules were performed using the 2023 version of the `GROMACS` software [15] with a time step of 0.5 fs. The simulation was run for 1.25 µs in the NVT ensemble using a cubic cell of 1.6 nm side length. The velocity rescale thermostat was used to maintain the temperature at 300 K, employing a stochastic factor with a time constant of 0.5 ps. A cutoff of 0.9 nm is used for

the Lennard-Jones interaction, which was then shifted at the cutoff. Long-range electrostatic interactions are handled using the smooth particle mesh Ewald method (SPME). From the trajectory, 250,000 time-uncorrelated configurations were saved, and from those configurations, 50, 100, 1000, and 10,000 configurations were randomly chosen, along with 1000 validation and 1000 test configurations. For all training sets, the validation and test sets were kept fixed. The MD simulations for the trained MACE models were performed with the Atomic Simulation Environment (ASE) MD suite. [16] Langevin NVT dynamics (T = 300 K) simulations were run for 1 ns with a timestep of 0.5 fs. Four separate simulations with different random seeds for initializing the Boltzmann-distributed velocities were performed. Snapshots from the simulations were saved at an interval of 100 timesteps. Bead configuration visualization was done with the `OVITO` software. [17]

[1] *Metatensor Models*, `https://github.com/lab-cosmo/metatensor-models`.

[2] M. J. Willatt, F. Musil and M. Ceriotti, *Phys. Chem. Chem. Phys.*, 2018, **20**, 29661–29668.

[3] D. P. Kingma and J. Ba, *arXiv preprint arXiv:1412.6980*, 2023.

[4] A. Paszke, S. Gross, F. Massa, A. Lerer, J. Bradbury, G. Chanan, T. Killeen, Z. Lin, N. Gimelshein, L. Antiga, A. Desmaison, A. Kopf, E. Yang, Z. DeVito, M. Raison, A. Tejani, S. Chilamkurthy, B. Steiner, L. Fang, J. Bai and S. Chintala, *Adv. Neural Inf. Process.*, 2019, **32**, 8024–8035.

[5] *Training a neural network on QM9*, `https://schnetpack.readthedocs.io/en/latest/tutorials/tutorial_02_qm9.html`.

[6] M. Geiger and T. Smidt, *arXiv preprint arXiv:2207.09453*, 2022.

[7] V. Blum, R. Gehrke, F. Hanke, P. Havu, V. Havu, X. Ren, K. Reuter and M. Scheffler, *Comput. Phys. Commun.*, 2009, **180**, 2175–2196.

[8] J. P. Perdew, A. Ruzsinszky, G. I. Csonka, O. A. Vydrov, G. E. Scuseria, L. A. Constantin, X. Zhou and K. Burke, *Phys. Rev. Lett.*, 2008, **100**, 136406.

[9] V. L. Deringer and G. Csányi, *Phys. Rev. B*, 2017, **95**, 094203.

[10] F. Musil, M. Veit, A. Goscinski, G. Fraux, M. J. Willatt, M. Stricker, T. Junge and M. Ceriotti, *The Journal of Chemical Physics*, 2021, **154**, 114109.

[11] W. C. Witt, C. van der Oord, E. Gelžinytė, T. Järvinen, A. Ross, J. P. Darby, C. H. Ho, W. J. Baldwin, M. Sachs, J. Kermode, N. Bernstein, G. Csányi and C. Ortner, *J. Chem. Phys.*, 2023, **159**, 164101.

[12] *Rascaline*, `https://github.com/Luthaf/rascaline`.

[13] K. K. Huguenin-Dumittan, P. Loche, N. Haoran and M. Ceriotti, *J. Phys. Chem. Lett.*, 2023, **14**, 9612–9618.

[14] J. L. F. Abascal and C. Vega, *J. Chem. Phys.*, 2005, **123**, 234505.

[15] H. Berendsen, D. van der Spoel and R. van Drunen, *Computer Physics Communications*, 1995, **91**, 43–56.

[16] A. H. Larsen, J. J. Mortensen, J. Blomqvist, I. E. Castelli, R. Christensen, M. Dułak, J. Friis, M. N. Groves, B. Hammer, C. Hargus, E. D. Hermes, P. C. Jennings, P. B. Jensen, J. Kermode, J. R. Kitchin, E. L. Kolsbjerg, J. Kubal, K. Kaasbjerg, S. Lysgaard, J. B. Maronsson, T. Maxson, T. Olsen, L. Pastewka, A. Peterson, C. Rostgaard, J. Schiøtz, O. Schütt, M. Strange, K. S. Thygesen, T. Vegge, L. Vilhelmsen, M. Walter, Z. Zeng and K. W. Jacobsen, *Journal of Physics: Condensed Matter*, 2017, **29**, 273002.

[17] A. Stukowski, *Modelling and Simulation in Materials Science and Engineering*, 2009, **18**, 015012.
